# Infective Endocarditis-Associated Cerebral Embolism With Successful Early Thrombectomy Followed by Subarachnoid Hemorrhage Six Hours Post-treatment: A Case Report

**DOI:** 10.7759/cureus.91648

**Published:** 2025-09-05

**Authors:** Yoshiaki Kakehi, Kozue Saito, Kana Hamada, Ichiro Nakagawa, Kazuma Sugie

**Affiliations:** 1 Neurology, Nara Medical University, Kashihara, JPN; 2 Neurosurgery, Nara Medical University, Kashihara, JPN

**Keywords:** cardiac embolism, endovascular thrombectomy, infective endocarditis, mechanical thrombectomy, subarachnoid hemorrhage

## Abstract

Infective endocarditis (IE)-associated cerebral embolism can lead to severe complications, including subarachnoid hemorrhage (SAH). Inflammation spreading from the embolus to the arterial wall may cause vascular fragility, increasing the risk of hemorrhage even after successful recanalization. Furthermore, mechanical stress during thrombectomy may intensify this fragility, potentially precipitating hemorrhage. However, the timing of the onset of these hemorrhagic complications remains unclear.

We report a 71-year-old man with IE who presented with sudden consciousness disturbance three months after discharge from antibiotic therapy. Imaging revealed left middle cerebral artery (MCA) occlusion with early ischemic changes. Thrombectomy was performed at three hours and 40 minutes after onset, achieving complete recanalization (expanded Thrombolysis in Cerebral Infarction, or eTICI 3). His neurological status initially improved, but six hours after surgery, he exhibited recurrent consciousness disturbance. A computed tomography (CT) scan revealed SAH in the left Sylvian fissure. No vascular anomalies or procedural complications were noted. This delayed event was likely due to a combination of vascular fragility from IE, inflammation spreading from the embolus, and mechanical stress from the procedure. Even with early and atraumatic recanalization, careful postoperative monitoring is essential.

## Introduction

Concurrent cerebral infarction and subarachnoid hemorrhage (SAH) in infective endocarditis (IE) significantly affect the prognosis of patients. A recent national cohort study showed that IE patients with SAH had markedly higher in-hospital mortality (OR 4.65) and a greater risk of acute ischemic stroke (OR 6.3), with 41.5% developing stroke during hospitalization versus 10.1% without SAH [1]. Endovascular treatment for cerebral embolism with IE is associated with a high risk of intracranial hemorrhagic complications, such as SAH, intracerebral hemorrhage, and rupture of infectious (mycotic) aneurysms [2-4]. Inflammation spreading from the emboli to the arterial walls, leading to arterial wall vulnerability, is the primary cause of SAH in these cases [5-9].

In this report, we present a case in which complete recanalization was achieved at three hours and 40 minutes after the onset of embolism; however, the patient still developed SAH six hours after the procedure. In IE-associated cerebral embolism, arterial wall fragility, caused by inflammation spreading from the embolus, may develop within a short time after the embolus impaction. Therefore, even if early thrombectomy is successfully performed, it is essential to remain vigilant regarding the risk of post-treatment SAH.

## Case presentation

**Figure 1:**
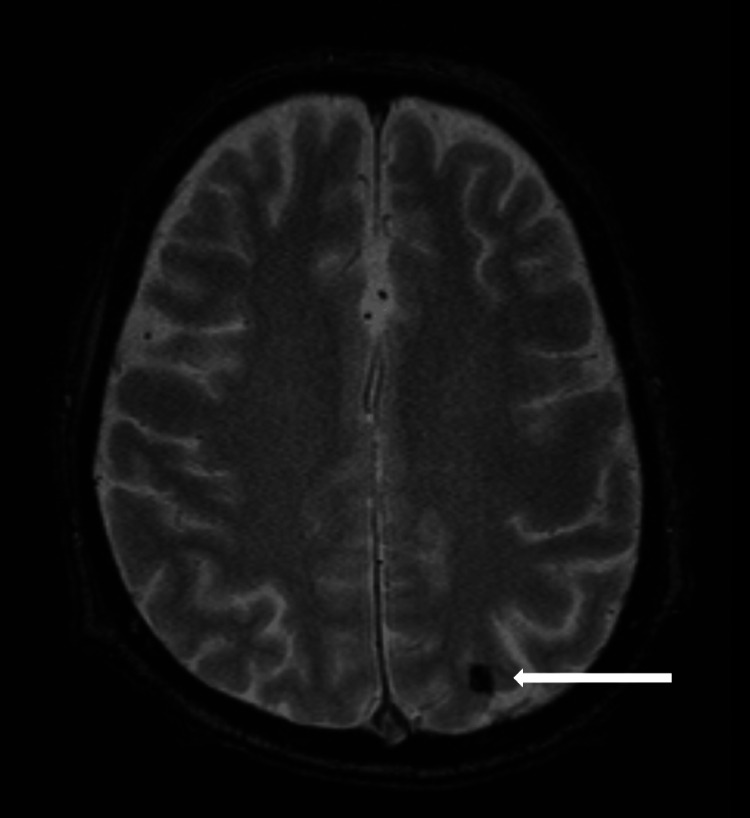
T2*-weighting effect on MRI A hypointense spot is observed in the left parietal lobe (white arrow). MRI, Magnetic resonance imaging

A 71-year-old male patient visited our hospital because of fever (>39°C). He was then diagnosed with sepsis caused by gram-positive cocci. Transesophageal echocardiography revealed vegetations on the right coronary cusp of the aortic valve and the anterior leaflet of the mitral valve. Thus, a diagnosis of IE was made. Antibiotic therapy with ampicillin-sulbactam/ceftriaxone was initiated. Although the vegetations did not resolve, surgery was considered too risky due to the patient’s overall condition. Multiple microbleeds were observed on magnetic resonance imaging (MRI), using the T2*-weighted sequence (Figure 1).

With the disappearance of inflammatory markers in blood tests, and sustained negative blood culture results, the patient was discharged home. Antibiotic therapy was discontinued after discharge.

**Figure 2:**
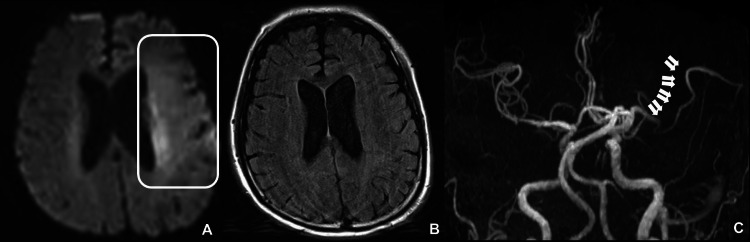
Initial MRI and MRA findings at admission (A) DWI reveals a faint high-signal area in the region of the left MCA (white box). (B) No signal changes are observed in the same area on FLAIR. (C) MRA shows occlusion of the left MCA (white arrows). MRI, Magnetic resonance imaging; MRA, Magnetic resonance angiography; DWI, Diffusion-weighted imaging; MCA, Middle cerebral artery; FLAIR, Fluid-attenuated inversion recovery

After three months, he was urgently transported to the hospital because of sudden altered consciousness. Upon admission, the patient’s vital signs were as follows: blood pressure, 117/59 mmHg; heart rate, 78 bpm (regular); oxygen saturation, 99% on room air; and body temperature, 38.0°C. A systolic heart murmur, similar to that observed during the previous hospitalization, was also noted. Furthermore, based on the Glasgow Coma Scale (GCS), his level of consciousness was E1V1M4. The neurological findings included left conjugate deviation, right facial paralysis, dysarthria, right-sided limb weakness and hypesthesia, right hemispatial neglect, and global aphasia. The patient’s National Institutes of Health Stroke Scale score was 24. Laboratory tests showed a mildly elevated white blood cell count (10,400/μL) and C-reactive protein level (2.76 mg/dL). The patient’s brain natriuretic peptide level was elevated to 153.3 pg/dL. The coagulation study results were normal. Arrhythmia was not detected on the electrocardiogram. Computed tomography (CT) scan did not show bleeding, early ischemic changes, or mass lesions, with an Alberta Stroke Program Early CT Score (ASPECTS) of 10/10. MRI revealed a faint high-signal area in the territory of the left middle cerebral artery (MCA) on diffusion-weighted imaging (DWI), with an ASPECTS+W score of 7/11. No high signal was observed on fluid-attenuated inversion recovery (FLAIR) imaging. Magnetic resonance angiography (MRA) found occlusion of the left MCA superior trunk (Figure 2). 

**Figure 3:**
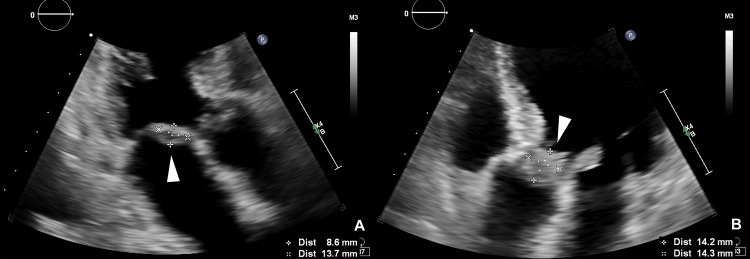
Initial TTE findings at admission (A-B) Vegetations are identified on both the mitral and aortic valves (white arrowheads). TTE, Transthoracic echocardiography

Transthoracic echocardiography (TTE) showed residual vegetations, consistent with those seen during the prior admission (Figure 3).

Blood cultures yielded *Staphylococcus lugdunensis*. These findings led to the diagnosis of IE-associated cerebral embolism.

**Figure 4:**
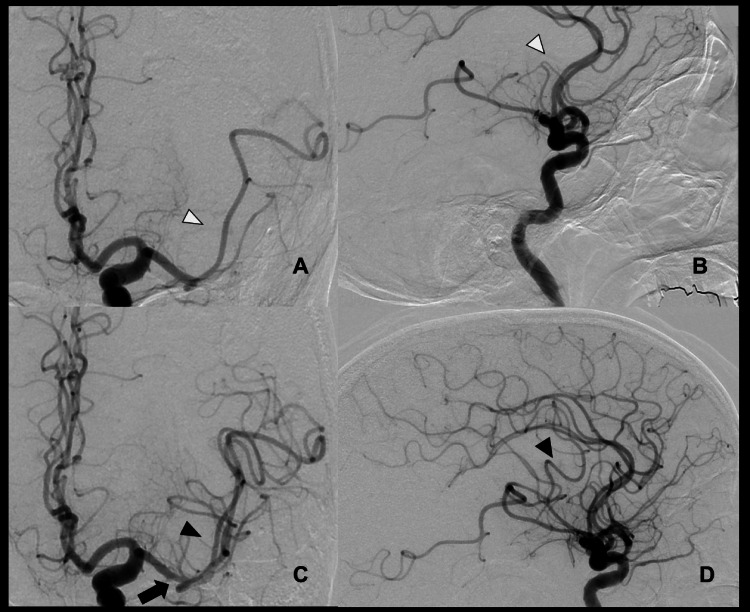
DSA findings pre- and post-thrombectomy (A-B) DSA shows occlusion of the left MCA M2 superior trunk in frontal and lateral views (white arrowheads). (C-D) Successful reperfusion (eTICI 3) was achieved after endovascular thrombectomy, as demonstrated in frontal and lateral views (black arrowheads). The M1 distal segment appeared slightly narrowed and irregular (black arrow). MCA, Middle cerebral artery; DSA, Digital subtraction angiography; eTICI, expanded Thrombolysis in Cerebral Infarction

Considering the presence of IE, recombinant tissue plasminogen activator therapy was considered inappropriate. The presence of a DWI-FLAIR mismatch on MRI suggested thrombectomy eligibility. From the right femoral artery, the 9-Fr Optimo 90-cm catheter (Tokai Medical Products, Aichi, Japan) and the 6-Fr JB2 125-cm Daimon catheter (Silux, Saitama, Japan) were coaxially inserted into the left internal carotid artery. Phenom27 160-cm (Medtronic, Irvine, CA, USA) and CHIKAI black14 200-cm (Asahi Intec, Tokyo, Japan) were advanced without traversing the lesion. Then, the 6F SOFIA™ FLOW 131-cm (MicroVention TERUMO, Aliso Viejo, CA, USA) was advanced with the direct aspiration first-pass technique. However, successful recanalization was not achieved. Subsequently, Phenom27 and CHIKAI black14 passed through the lesion. Then, the 6F SOFIA™ FLOW was advanced to the proximal end of the thrombus, followed by the direct aspiration first-pass technique, which resulted in complete recanalization. The onset-to-reperfusion time was three hours and 40 minutes. After thrombectomy, the MCA-M1 distal segment appeared slightly narrowed and irregular compared with the preoperative angiogram. Considering that a relatively large 6F SOFIA catheter was used in proportion to the vessel diameter, stress on the vessel wall cannot be excluded (Figure 4). 

During the thrombectomy procedure, no intravenous anticoagulants, including heparin, were administered in consideration of the underlying IE. Dyna CT was performed immediately after mechanical thrombectomy, but the image quality was poor, and hemorrhagic complications could not be evaluated. No apparent dissection or aneurysm formation was observed on post-recanalization digital subtraction angiography (DSA), and, given the favorable clinical course, we judged that there was no major hemorrhagic complication.

**Figure 5:**
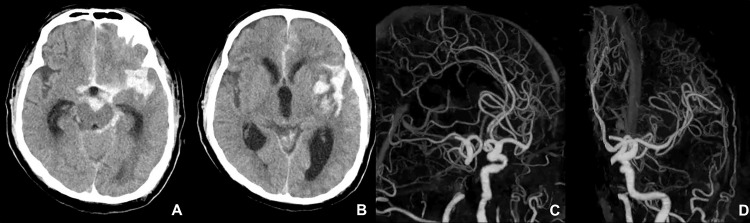
CT and CT angiography findings six hours after thrombectomy (A-B) CT reveals SAH extending from the interhemispheric fissure to the left Sylvian fissure, subcortical hemorrhage in the left insular cortex, and infarction in the left MCA territory. (C-D) CT angiography did not show any vascular abnormalities indicative of the bleeding source (C: right anterior oblique view; D: left anterior oblique view). CT, Computed tomography; SAH, Subarachnoid hemorrhage

Following recanalization, systolic blood pressure was maintained below 140 mmHg without the need for antihypertensive agents. Given that embolism is associated with IE, antithrombotic therapy was not administered postoperatively. The pathological findings of the retrieved thrombus revealed red blood cells, fibrin, platelets, and neutrophils. In addition, *S. lugdunensis* was identified in the thrombus culture, supporting the diagnosis of an embolus originating from IE. Improvements in the level of consciousness (GCS E3V1M5) and left eye deviation were observed immediately after thrombectomy. At six hours postoperatively, the patient exhibited sudden recurrence of consciousness disturbance (GCS E1V1M4) and left gaze deviation, accompanied by an elevation of systolic blood pressure exceeding 180 mmHg. CT scan revealed an SAH in the left Sylvian fissure, with intracerebral hemorrhage and infarction in the left MCA territory. CT angiography did not reveal any vascular anomalies that could serve as sources of bleeding (Figure 5). 

**Figure 6:**
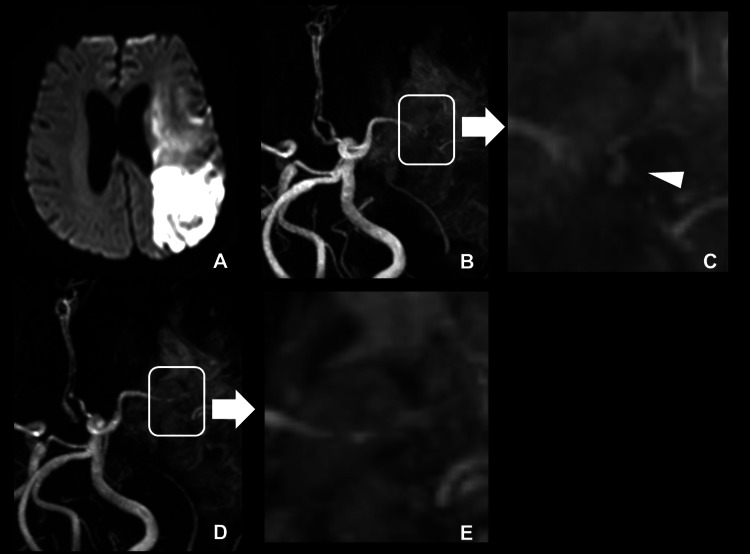
Follow-up MRI and MRA findings on day 18 and day 28 (A) Follow-up MRI performed on day 18 revealed progression of cerebral infarction in the left MCA territory. (B) MRA on the same day showed severe vasospasm and a suspected pseudoaneurysm in the left MCA (white box). (C) Enlarged view of the area marked by the white box in panel B, demonstrating the suspected pseudoaneurysm (white arrowhead). (D) Follow-up MRA on day 28 demonstrated disappearance of the pseudoaneurysm (white box). (E) Enlarged view of the area marked by the white box in panel D, demonstrating no residual pseudoaneurysm. MRI, Magnetic resonance imaging; MRA, Magnetic resonance angiography; MCA, Middle cerebral artery

A follow-up CT performed the next day showed no progression of the hematoma or SAH. As there was no indication for surgery, conservative management was chosen, and intravenous vancomycin (VCM) therapy was initiated. Fever resolved after the initiation of VCM; however, TTE performed on hospital day 10 revealed an increase in vegetation size, and VCM administration was continued. On hospital day 18, follow-up TTE showed no further growth of the vegetation, and VCM was discontinued on day 38. Thereafter, blood cultures remained negative, and no increase in vegetation size was observed on subsequent echocardiographic evaluations. Follow-up MRI and MRA performed on day 18 revealed the progression of cerebral infarction due to severe cerebral vasospasm and a suspected pseudoaneurysm in the left MCA. Surgical intervention for the aneurysm was deemed too risky, given the patient's overall condition; therefore, antibiotic therapy was continued. The pseudoaneurysm resolved on follow-up MRA performed on day 28 (Figure 6). 

The patient had a modified Rankin scale score of 5 and was transferred to a rehabilitation hospital.

## Discussion

In this report, we present a case of cerebral embolism that occurred after the remission of IE. Concurrent cerebral infarction and SAH in IE significantly affect patient prognosis [1]. Despite achieving complete recanalization via thrombectomy within three hours and 40 minutes, the patient developed SAH from the occlusion site six hours after the procedure. SAH is a common complication of IE, and arterial wall fragility caused by inflammation spreading from the embolus is a proposed mechanism. As these changes can develop within an extremely short time after embolus impaction, patients are still at risk of SAH even after early and successful thrombectomy.

Recent data from a large international cohort showed that stroke occurred in about 17% of IE patients, with in-hospital mortality of 17.7%. These findings highlight the frequent and severe cerebrovascular complications in IE [10]. In particular, a recent national cohort study showed that IE patients complicated by SAH had markedly higher in-hospital mortality and an increased risk of acute ischemic stroke, compared with those without SAH [1]. Pathological studies have suggested that vessel wall fragility at embolic sites may contribute to rupture in IE [9]. These epidemiological and pathological insights highlight that the cerebrovascular complications of IE are not only frequent, but also carry grave prognostic implications, underscoring the clinical importance of the present case. In the differential diagnosis of embolic stroke with hemorrhagic complications, it is also important to consider marantic (nonbacterial thrombotic) endocarditis, which can mimic IE and carries a high risk of embolic events [11].

Thrombectomy for IE-associated cerebral embolism is associated with a high risk of SAH. Moreover, the clinical outcomes of patients with IE undergoing thrombectomy have been reported to be generally poor [2]. However, in recent years, some reports have shown that thrombectomy is effective in selected cases [3,4]. In particular, Feil et al. revealed a negative association between the rate of cerebral arterial reperfusion and IE-related mortality [3]. In contrast, Mowla et al. emphasized that caution should be observed, as SAH can develop after thrombectomy. They further suggested that this complication may result from weakening of the arterial wall due to inflammatory infiltration caused by the embolus [4]. Previous autopsy reports of cases in which IE was complicated by cerebral embolism showed inflammatory cell infiltration into the arterial wall, and disruption of the internal elastic lamina at the embolic site [5-8].

In our case, complete recanalization was achieved three hours and 40 minutes after onset, leading to clinical improvement. However, the patient developed SAH six hours after the procedure, and his modified Rankin scale score was 5. Despite the confirmation of negative blood culture results, vegetation was noted before the onset of cerebral embolism. The hypointense spots on T2*-weighted MRI during the previous hospitalization may reflect mycotic aneurysms or microbleeds, and these findings indicate underlying vascular fragility related to IE [12,13]. Additionally, the thickest hematoma was observed in the left Sylvian fissure, suggesting that, as reported in previous autopsy cases [5-8], inflammation spreading from the embolus lodged in the left MCA may have further weakened the arterial wall. Although the procedure was performed carefully, with no evidence of perforation by the micro-guidewire or micro-catheter, the relatively large diameter of the aspiration catheter, in proportion to the vessel size, may have imposed mechanical stress. The slight narrowing and irregularity of the distal MCA seen immediately after thrombectomy further support this possibility. Thus, delayed intracranial hemorrhage may still occur due to a combination of procedure-induced mechanical stress, underlying fragility, and embolus-related inflammation - even in cases where complete thrombus retrieval is achieved without apparent complications. The pseudoaneurysm observed on follow-up MRA supports the likelihood of this pathological combination.

Marnat et al., Feil et al., and Mowla et al. reported a high incidence of intracranial hemorrhagic complications associated with endovascular treatment for cerebral embolism due to IE; however, the timing of hemorrhage onset was not specified in their studies [2-4]. Table 1 shows a summary of cases of cerebral embolism associated with IE in which the site of occlusion was identified and SAH occurred, as in the current case. Cases in which the timing of SAH was unclear, cases in which the occluded vessel and hemorrhage site were distant, and cases in which SAH occurred as a complication of thrombectomy were excluded from the analysis [5,6,14-22].

**Table 1:** Twelve cases of cerebral embolism associated with IE, in which the site of occlusion was identified and SAH occurred IE, Infective endocarditis; SAH, Subarachnoid hemorrhage; M, Male; F, Female; ICH, Intracranial cerebral hemorrhage; BA, Basilar artery; MCA, Middle cerebral artery; ACom, Anterior communicating artery; ACA, Anterior cerebral artery; eTICI, expanded Thrombolysis in Cerebral Infarction; mRS, modified Rankin scale

Authors/year	Sex/age	Occlusion site	Cerebral hemorrhage	Time to hemorrhagic complication	Source of infection	Imaging findings of the bleeding point	Thrombectomy and the eTICI score	Patient outcome
Yock (1984) [5]	M/27	BA	SAH	1.5 days	IE	Unavailable	Not performed	Death, mRS score of 6
Krapf et al. (1999) [14]	F/56	Right MCA	SAH	1.5 days	IE	Unavailable	Not performed	Hemiparesis
Yamaguchi et al. (2004) [15]	F/56	Right MCA	Subcortical hematoma	35 days	IE	Pseudoaneurysm on the M2 branch	Not performed	Hemiparesis
Inoue et al. (2006) [16]	M/38	Left MCA	SAH	4 days	Unavailable	Pseudoaneurysm on the M2 branch	Not performed	Hemiparesis
Allen et al. (2012) [17]	M/58	Right ACA	Subcortical hematoma	1 day	IE	Unavailable	Not performed	Death, mRS score of 6
Sorkin et al. (2013) [18]	F/14	Right MCA	ICH	5 days	IE	Pseudoaneurysm on the M1 branch	Not performed	Hemiparesis
Saito et al. (2014) [6]	F/78	Right MCA	SAH	2 days	Unavailable	Unavailable	Not performed	Death, mRS score of 6
Wang et al. (2017) [19]	M/25	Right MCA	SAH	1.5 days	Unavailable	Pseudoaneurysm on the M2 branch	Not performed	Dysarthria, left facial droop, left upper extremity weakness
Nukata et al. (2022) [20]	M/58	Left ACA	Subcortical hematoma	1.5 days	IE	Pseudoaneurysm on A4	Not performed	Aphasia and hemiparesis, mRS score of 5
Yanagawa et al. (2023) [21]	F/66	Left MCA	Subcortical hematoma and SAH	3 days	IE	Pseudoaneurysm on the M1/2 bifurcation	Not performed	Death, mRS score of 6
Kurauchi et al. (2024) [22]	M/70	ACom to bilateral ACA	SAH	3 days	IE	Pseudoaneurysm on ACom	eTICI 2b50	Death, mRS score of 6
Current case (2025)	M/71	Left MCA	SAH	1 day	IE	Unavailable	eTICI 3	Aphasia and hemiparesis, mRS score of 5

In all cases, SAH developed by the fifth postoperative day. In a pathological study by Masuda et al., the arterial wall at embolic sites demonstrated inflammatory cell infiltration, medial necrosis, and disruption of the internal elastic lamina in patients with IE. In several cases, arterial rupture was observed adjacent to the emboli, suggesting that inflammation spreading from the embolus into the vessel wall may have contributed to the hemorrhagic event. Notably, no pseudoaneurysms were identified in these ruptured arteries, indicating that direct vessel wall injury and subsequent hemorrhage can occur in the absence of aneurysmal formation [9]. These findings support the hypothesis that, even without angiographic abnormalities - such as in our case - embolus-induced inflammation can rapidly compromise the structural integrity of the arterial wall, potentially leading to delayed intracranial hemorrhage.

In the present case, SAH occurred six hours after a successful mechanical thrombectomy that achieved complete recanalization. This complication likely resulted from the combined effects of vascular fragility associated with IE, mechanical stress during thrombectomy, and inflammatory infiltration from the embolus. The time delay before hemorrhage onset may reflect the interval required for embolus-induced inflammation to compromise the arterial wall, ultimately leading to vessel rupture. Although previous autopsy cases [5-8] and the report by Masuda et al. [9] have described embolus-related inflammatory extension into the arterial wall, the precise timeframe of this process has not been clearly established. In light of the present case, arterial wall injury due to embolus-induced inflammation may develop within only a few hours after embolic impaction, and such propagation of inflammation might not be prevented even when complete thrombus retrieval is achieved. Although pathological confirmation was not obtained, the presence of hypointense spots on T2*-weighted MRI suggested underlying vascular fragility related to IE. In addition, post-recanalization DSA demonstrated slight narrowing and irregularity of the distal MCA-M1 segment compared with the preoperative angiogram, which may suggest mechanical stress from the thrombectomy. Furthermore, follow-up MRI revealed a pseudoaneurysm at the site where the embolus had lodged, which may indicate embolus-induced arterial wall inflammation. Therefore, in cases of cerebral infarction associated with IE, careful postoperative monitoring and heightened vigilance for hemorrhagic complications are essential, even after successful thrombectomy.

## Conclusions

We report a rare case of IE-associated cerebral embolism complicated by SAH occurring six hours after successful thrombectomy. This delayed hemorrhage likely resulted from a combination of vascular fragility associated with IE, mechanical stress during thrombectomy, and embolus-induced inflammatory infiltration into the arterial wall, as suggested by imaging findings. The short interval before hemorrhage onset suggests that such inflammatory processes can progress rapidly - potentially within only a few hours. This case is unique in demonstrating that, even after successful thrombectomy, hemorrhagic complications such as SAH may develop in the acute phase, highlighting the need for vigilant postoperative monitoring in IE-associated cerebral embolism.
